# Regulation of Immune Cells by Eicosanoid Receptors

**DOI:** 10.1100/tsw.2007.181

**Published:** 2007-09-01

**Authors:** Nancy D. Kim, Andrew D. Luster

**Affiliations:** Center for Immunology and Inflammatory Diseases, Division of Rheumatology, Allergy, and Immunology, Massachusetts General Hospital, Harvard Medical School, Boston, USA

**Keywords:** prostaglandin D_2_, DP_1_, DP_2_, CRTH_2_, leukotriene B_4_, BLT_1_, BLT_2_, cysteinyl leukotriene, CysLT_1_, CysLT_2_

## Abstract

Eicosanoids are potent, bioactive, lipid mediators that regulate important components of the immune response, including defense against infection, ischemia, and injury, as well as instigating and perpetuating autoimmune and inflammatory conditions. Although these lipids have numerous effects on diverse cell types and organs, a greater understanding of their specific effects on key players of the immune system has been gained in recent years through the characterization of individual eicosanoid receptors, the identification and development of specific receptor agonists and inhibitors, and the generation of mice genetically deficient in various eicosanoid receptors. In this review, we will focus on the receptors for prostaglandin D_2_, DP_1 _and DP_2_/CRTH2; the receptors for leukotriene B_4_, BLT_1_ and BLT_2_; and the receptors for the cysteinyl leukotrienes, CysLT_1_ and CysLT_2_, by examining their specific effects on leukocyte subpopulations, and how they may act in concert towards the development of immune and inflammatory responses.

## INTRODUCTION

Eicosanoids comprise a family of 20-carbon–based lipid mediators derived from arachidonic acid, possessing multiple diverse functions on cells of the immune system. Upon liberation from the cell membrane by cytosolic phospholipase $A_2$ ($PLA_2$), arachidonic acid (AA) is rapidly metabolized into two major classes of eicosanoids, prostaglandins and leukotrienes, both of which are associated with numerous inflammatory, infectious, and ischemic processes. While the production and regulation of these lipid mediators are discussed in a separate paper in this issue[1], this review will outline their major effects on cellular subpopulations of the immune system through their specific receptor usage. In allergic and inflammatory conditions, levels of prostaglandin $D_2$, leukotriene $B_4$, and the cysteinyl leukotrienes $LTC_4$, $LTD_4$, and $LTE_4$ are linked to heightened leukocyte infiltration into affected tissues. Recent studies defining how these lipid mediators orchestrate complex pathways of leukocyte migration, degranulation, and mediator release through their specific receptor usage will be reviewed.

## THE PROSTAGLANDIN RECEPTORS $DP_1$ AND $DP_2$/CRTH2

Prostaglandin $D_2$ ($PGD_2$) is the predominant prostaglandin generated by activated mast cells and has been long associated with inflammation and allergic responses, as demonstrated by elevated levels of $PGD_2$ within bronchoalveolar lavage fluids[2] and in allergic skin biopsies[3] of antigen-challenged patients. $DP_1$, the first identified receptor for $PGD_2$, is a prostanoid G-protein coupled–type receptor with 73% homology at the amino acid level between the human and mouse forms[4]. $DP_1$ preferentially binds the ligand $PGD_2$ with a $K_d$ of 1.5 n*M*, in addition to the selective $DP_1$ agonist BW245C and $DP_1$ antagonist BWA868C with equal affinities, and binds with 100-fold or less affinity to $PGE_2$, $PGF_{2\alpha}$, iloprost, and the thromboxane $A_2$ mimetic U46619. Binding of $DP_1$ by $PGD_2$ results in the elevation of intracellular cyclic AMP (cAMP) levels and $Ca^{2+}$ mobilization, but not the production of inositol 1,4,5-triphosphate ($IP_3$)[5]. In organ tissues, $DP_1$ is expressed at low levels in the small intestine and retina in humans[5], while in mice, $DP_1$ is found in the ileum, lung, stomach, and uterus[6]. In the original description of human $DP_1$, basophils were the only human leukocytes found to express $DP_1$, but since then, $DP_1$ message has been demonstrated in human eosinophils[7], monocytes, dendritic cells[8], and Th1 and Th2 cells[9] at low levels. In mice, monocytes, dendritic cells[8], and eosinophils[10] express $DP_1$. In an ovalbumin (OVA)-induced murine model of asthma, mice deficient in the $DP_1$ receptor demonstrated decreased levels of eosinophils and lymphocytes, and the cytokines IL-4, IL-5, and IL-13, suggesting that $DP_1$ plays a critical role in the induction of Th2-mediated allergic responses[11] (Table 1).

**TABLE 1**
**$DP_1$ and $DP_2$/CRTH2 Receptors**

| | $DP_1$ | $DP_2$/CRTH2 |
|---|---|---|
| Receptor expression on leukocytes | *Human*: eosinophils, basophils, monocytes, dendritic cells, Th1 and Th2 cells<br>*Mouse*: monocytes, dendritic cells, eosinophils | *Human*: Th2 cells, type 2 cytotoxic cells, basophils, eosinophils, monocytes<br>*Mouse*: Th1 and Th2 cells, eosinophils, monocytes, mast cell |
| Agonists | $PGD_2$ = $PDJ_2$<br>BW245C<br>ZK110841<br>SQ27986 | $PGD_2$ = $PGJ_2$ = 15d-$PGD_2$ =<br>DK-$PGD_2$>$\Delta^{12}$-$PGJ_2$<br>indomethacin |
| Antagonists | BWA868C<br>AH6809 | BM7 (neutralizing antibody)<br>Ramatroban (BAY-u3405) |

However, certain data suggested that $PGD_2$ might activate leukocytes through an alternate receptor to $DP_1$. In the OVA-induced asthma model, $DP_1$ up-regulation in the lung primarily occurs on the airway epithelium, and serum IgE levels and Th2 cytokine production by antigen-stimulated splenocytes from $DP_1$-deficient mice is preserved, indicating intact immune responses[11]. Activation of $DP_1$ through $G_{s\alpha}$ proteins results in an increase of intracellular cAMP typically associated with dampening of cellular effector function, contrary to the immune cell activation observed in $PGD_2$-high states, such as asthma and allergic responses. In addition, $PGD_2$ induces eosinophil chemotaxis, CD11b expression, and L-selectin expression in a manner neither replicated by the $DP_1$ receptor agonist BW245C nor inhibited by $DP_1$ receptor antagonist BWA868C[12], leading investigators to postulate that there existed a separate $PGD_2$ receptor whose proinflammatory functions were diametrically opposed to the suppressive effects of $DP_1$ activation.

In 1999, Nagata et al. described a novel, cell surface molecule expressed in Th2 cells,[13] eosinophils, and basophils, which they named CRTH2 (chemoattractant receptor-homologous molecule expressed on Th2 cells) that responded to a mast cell–derived factor. Subsequently, they identified $PGD_2$

as the mast cell–derived ligand for CRTH2, which is now also known as $DP_2$. Unlike $DP_1$ and the other prostanoid receptors, $DP_2$ phylogenetically shares more characteristics with the formyl peptide-like receptors (FPRs), a group of receptors that includes the chemoattractant C5a receptor and the leukotriene receptors $BLT_1$ and $CysLT_1$[14]. Activation of $DP_2$ leads to depression of intracellular cAMP levels, $Ca^{2+}$ mobilization, and generation of diacylglycerol (DAG) and $IP_3$ through $G_i$ protein signaling[15]. $DP_2$ mRNA is ubiquitously expressed in various human tissues, including the brain, heart, intestine, thymus, spleen, liver, and small and large intestine[16], while in the mouse, $DP_2$ mRNA is found at low levels in the lung, liver, kidney, brain, heart, thymus, and spleen[17]. In human peripheral blood leukocytes, $DP_2$ is found on Th2 cells, type 2 cytotoxic T (Tc2) cells, basophils, eosinophils, and in low quantities on monocytes, but not on Th1 cells, CD $19^+$ B cells, NK cells, immature dendritic cells, or neutrophils[8,16]. In sharp contrast, mouse $DP_2$ is expressed on both Th1 and Th2 cellular subsets[17], in addition to eosinophils and monocyte, mast cell, and B cell lines[10] (Table 1).

## $DP_1$ and $DP_2$ and T Cells

The $DP_2$ receptor was first identified as an orphan G-protein coupled chemoattractant receptor selectively expressed on human Th2 cells[13]. In one study characterizing human peripheral blood mononuclear cells (PBMCs), a small percent of $CD4^+$ T cells (0.4–6.5%) and $CD8^+$ T cells (3.5%) were $DP_2$ -positive, with the majority of the $DP_2$ -positive $CD4^+$ T cells being activated effector/memory T cells as indicated by $CD45RA^-$, $CD45RO^+$, and $CD25^+$ staining[13]. Subsequent studies confirmed that $DP_2$ identifies Th2 and Tc2 cells more specifically than either CCR3 or CCR4, two other chemoattractant receptors preferentially expressed on Th2 cells[18]. In keeping with known chemoattractant properties of its FPR structure, $DP_2$ activation by $PGD_2$ and the $DP_2$-specific agonist 13,14-dihydro-15-keto $PGD_2$ (DK-$PGD_2$) induces chemotaxis[14] and CD11b up-regulation[9] in Th2 cells, while the DP1 agonist BW245C does not. $PGD_2$ induces Th2 cell production of IL-4, IL-5, and IL-13, a result that is replicated to a lesser extent by the DK-$PGD_2$, but not BW245C, indicating activity through $DP_2$[9,19]. These results demonstrate that $DP_2$ amplifies Th2 cell responses by inducing their migration, enhancing their adhesiveness to endothelial surfaces by increasing CD11b surface expression, and invoking further elaboration of Th2 cytokines.

Although early studies did not demonstrate significant $DP_1$ expression in human T cells[14], addition of $PGD_2$ or the $DP_1$ agonist BW245C to CD3/CD28-stimulated CD4 and CD8 cells isolated from PBMCs results in decreased interferon (IFN)-$\gamma$ and IL-2 production, while the $DP_2$ agonist DK-$PGD_2$ has no effect on the production of these cytokines, indicating this $PGD_2$ effect is mediated through selective $DP_1$ activation[9]. In addition, selective $DP_1$ stimulation decreases the number of IL-4-secreting Th2 cells in a dose-dependent manner, but has no discernible effect on IL-2, IL-5, or IL-13 production. On increasing the numbers of cycles in reverse transcription polymerase chain reaction (RT-PCR), $DP_1$ mRNA becomes detectable in both Th1 and Th2 cells at low levels[9]. However, competitive binding assays show that $DP_2$ is the dominant receptor on Th2 cells by preferentially binding $PGD_2$ over DP1[19]. Therefore, these studies demonstrate that $DP_1$ may provide a counter-regulatory effect on Th2 cells in opposition to $DP_2$, suggesting that $DP_2$ antagonists may actually further suppress Th2 responses not only by blocking $DP_2$, but also by increasing the availability of $PGD_2$ to bind $DP_1$ that would otherwise activate $DP_2$.

Although mouse $DP_2$ shares significant homology with the human $DP_2$ receptor, a significant distinction between the two species is the fact that $DP_2$ is expressed on both Th1 and Th2 subsets in mice[17], suggesting that a strict dichotomy between $DP_2$ regulation of Th1 and Th2 subsets may not exist in this species. Surprisingly, $DP_2^{-/-}$ mice on a C57Bl/6 background develop increased eosinophilia in an OVA-induced model of asthma, and *in vitro* CD3/CD28 stimulation of T cells from $DP_2^{-/-}$ mice generates significantly more IL-5 than wild-type T cells[20], suggesting that $DP_2$ may actually play an anti-inflammatory role in mice by suppressing T-cell production of Th2 cytokines in mice. $DP_2^{-/-}$ mice on both C57Bl/6 and Balb/c backgrounds generated by a separate group reportedly do not display significant differences in pulmonary infiltration in an asthma model nor in splenocyte production of IL-4, IL-5, or IFN-$\gamma$ compared with wild-type mice[21]. While Balb/c $DP_2^{-/-}$ mice exhibit decreased swelling and leukocyte infiltration in an IgE-mediated cutaneous reaction model, this clinical finding is not associated with significant changes in IL-4 or IFN-γ production[21]. These results contrast previous studies in which administration of the $DP_2$ selective agonist DK-$PGD_2$ exacerbates mouse models of asthma and atopic dermatitis[10], which instead suggest a proinflammatory role for murine $DP_2$.

These collective studies clearly indicate a dominant role for $DP_2$ in the direct activation of Th2 cells in humans. Although data suggest that $DP_1$ activation of T cells may play a counter-regulatory role by suppressing cytokine production and inhibiting leukocyte movement, the precise physiological relevance of $DP_1$ receptor activation on human Th1 and Th2 cells still remains to be defined more fully, requiring further dedicated studies of $DP_1$ activation on separate Th1 and Th2 cellular populations. In mice, the role of $DP_2$ activation on T cells and its role in disease pathogenesis is less well delineated, with the coincident existence of $DP_2$ on both Th1 and Th2 populations and conflicting phenotypes in allergic disease models, indicating that $DP_2$ may not play as critical a role in inducing Th2 responses in mice as in humans.

## $DP_1$ and $DP_2$ in Eosinophils and Basophils

$PGD_2$ is a major eicosanoid product of mast cells, and levels of $PGD_2$ are elevated in allergic responses, suggesting an important role for $PGD_2$ in eosinophil activation. Addition of $PGD_2$ to eosinophils induces a number of proinflammatory responses, including chemokinesis, degranulation, and leukotriene production. Both $PGD_2$ receptors, $DP_1$ and $DP_2$, are expressed on human basophils[14] and eosinophils[7,22]. Through the use of $DP_1$- and $DP_2$-specific agonists, $DP_2$ has been identified as the critical receptor for eosinophil chemokinesis, chemotaxis, CD11b up-regulation, and degranulation, while selective $DP_1$ activation inhibits eosinophil apoptosis in culture conditions[7,12]. $PGD_2$ also induces similar responses in basophils through $DP_2$ activation, although $DP_1$ stimulation does not have antiapoptotic effects in this population[23]. Pretreatment of eosinophils with $PGD_2$ enhances their chemotaxis towards eotaxin-1 and 5-oxo-6,8,11,14-eicosatetraenoic acid (5-oxo-ETE) through activation of the $DP_2$ receptor, while $PGD_2$ activation of $DP_2$ suppresses basophil chemotaxis to these agents, indicating that $DP_2$ activation may preferentially augment eosinophil migration towards other subsequently encountered chemoattractants[24]. Therefore, although $DP_1$ and $DP_2$ are coexpressed on eosinophils and bind to $PGD_2$ with roughly equal affinities[14], $DP_2$ appears to exert a dominant proinflammatory effect on these cells. This effect may be explained, in part, by the relatively low expression of $DP_1$ on eosinophils compared with $DP_2$, which may up-regulated under inflammatory conditions[15].

$PGD_2$ is short lived *in vivo*, but its metabolic and degradation products also retain specific biologic activities that have been studied in eosinophils, shedding further light on selective $DP_1$ and $DP_2$ activation. $PGJ_2$ is the initial dehydration product of $PGD_2$, which can then be further metabolized to 15-dexoxy-$\Delta^{12,14}$-$PGJ_2$ (15d-$PGJ_2$) and $\Delta^{12}$-$PGJ_2$, all of which are found in blood plasma incubated with $PGD_2$[25]. While $PGJ_2$ binds to both $DP_1$ and $DP_2$, 15d-$PGD_2$ selectively binds $DP_2$ over $DP_1$[14]. At low concentrations ($10^6$–$10^9$ *M*), 15d-$PGJ_2$ induces eosinophil calcium flux, actin polymerization, and CD11b expression, while it has no effect on $DP_1$-mediated platelet aggregation[26]. These results contrast previous findings that 15d-$PGJ_2$ (at micromolar concentrations) possesses anti-inflammatory effects by inhibiting monocyte cytokine production and release through activation of peroxisome proliferator-activated receptor-γ (PPARγ)[27]. $\Delta^{12}$-$PGJ_2$ also induces chemotaxis and respiratory burst in eosinophils, as well as enhances eosinophil movement to CCL11/eotaxin-1 and mobilization from the bone marrow[28]. Although $\Delta^{12}$-$PGJ_2$ binds with weak affinity to both $DP_1$ and $DP_2$[14], $DP_1$ activation does not lead to significant calcium flux or chemotaxis in eosinophils, leading the authors to hypothesize that these proinflammatory $\Delta^{12}$-$PGJ_2$–mediated effects on eosinophils occur through $DP_2$ stimulation rather than $DP_1$.

In mice as in humans, both $DP_1$ and $DP_2$ are expressed on eosinophils. In *in vitro* chemotaxis studies, $PGD_2$ induces migration of eosinophils by activation of $DP_2$, while $DP_1$ activation has no such effect.

Addition of the $DP_2$ agonist DK-$PGD_2$ increases organ eosinophilia in models of OVA-induced asthma and atopic dermatitis in mice[10], suggesting that $DP_2$ contributes to eosinophil infiltration in allergic disease by inducing their movement into inflamed tissues. However, replication of the asthma model in mice lacking the two $PGD_2$ receptors has paradoxically yielded opposite results. $DP_1^{-/-}$ mice have decreased airway eosinophilia in an OVA-induced asthma model[11], while $DP_2^{-/-}$ mice display either no difference in lung leukocyte infiltration or increased airway eosinophilia in separate studies[20,21]. An IgE-mediated inflammatory cutaneous reaction is suppressed in $DP_2^{-/-}$ mice, however, with significantly decreased ear swelling and dermal leukocyte infiltration, including lymphocytes, eosinophils, neutrophils, and mast cells[21].

These data suggest that the role of $PGD_2$ in inducing eosinophilic inflammation in mice occurs by mechanisms more complex than directly inducing eosinophil activation and movement. Although *in vitro* studies show that $DP_2$ is capable of directly activating eosinophils, *in vivo* $PGD_2$-mediated allergic effects may depend on cooperation of resident cells that are organ -or site-specific. In murine lungs, $DP_1$ is primarily expressed on the epithelial cells of the bronchioles and type II alveolar epithelial cells, with lower expression in the type I alveolar epithelial cells and leukocytes[11]. It is hypothesized that $DP_1$-activated airway epithelium is required for production of cytokines and chemokines, leading to Th2 cell and eosinophil recruitment into the lung. Therefore, in murine pulmonary allergic reactions, $PGD_2$ may play a predominant role by activating $DP_1$ receptors on epithelium and generating eosinophil chemoattractants other than $PGD_2$, which would be consistent with the findings that $DP_2^{-/-}$ mice do not exhibit reduced inflammation.

## $DP_1$ and $DP_2$ in Dendritic Cells

$DP_1$ and $DP_2$ are both expressed on murine dendritic cells[29,30]. A role for $PGD_2$-mediated signaling in dendritic cells was first discovered through studies of a murine parasitic infection model. In mice, percutaneous infection with the helminth *Schistosoma mansoni* led to retention of Langerhans cells (LC), specialized antigen-presenting cells, within the epidermis, rather than migration to the skin-draining lymph nodes as would normally occur after infection. It was found that helminth infection led to $PGD_2$ production in the skin and retention of LCs within the skin through $DP_1$ receptor activation[29]. Further studies showed that *S. mansoni* produces $PGD_2$ through the parasitic enzyme Sm28GST, which shares 32% homology with hematopoietic-type PGD synthase[31], implying that parasite generation of $PGD_2$ and subsequent inhibition of dendritic cell migration through $DP_1$ activation evolved as a means for parasites to evade the immune system. Indeed, $DP_1$-deficient mice exhibited disappearance of LC from the epidermis and decreased worm and egg burden after infection, demonstrating that this parasitic evasion process is thwarted in the absence of $DP_1$ signaling[31].

Subsequently, similar roles for $DP_1$ regulation of dendritic cells have been found in other inflammation models. In a mouse contact hypersensitivity model, pretreatment with the $DP_1$ agonist BW24C leads to decreased ear swelling and epidermal LC retention, rather than migration to the skin-draining lymph nodes, after topical FITC application[29]. Similarly, in an OVA-induced model of atopic dermatitis, $DP_1$ activation results in increased LC epidermal retention and decreased numbers of activated OVA-specific T cells in affected skin, with resultant decreases in epidermal swelling[32]. Cytokine and chemokine analysis of the skin showed that $DP_1$ activation leads to a down-regulation of Th2-associated markers (IL-4, CCL11/eotaxin-1, CCL22/MDC, CCL17/TARC, CCR3, and CCR4) along with increased levels of Th1-associated markers (IFN-$\gamma$, CXCL10/IP-10, CXCR3, and CCR5)[32]. In the lung, $DP_1$, but not $DP_2$, activation leads to decreased lung dendritic cell migration to the thoracic lymph nodes after intratracheal instillation of FITC-OVA, as well as decreased lymph node T-cell proliferation and IL-4, IL-10, and IFN-$\gamma$ production[33].

Although mRNA for both $DP_1$ and $DP_2$ are found in human dendritic cells, flow cytometry analysis demonstrates significant expression of only $DP_1$ on both immature and mature DCs[8]. Both TNF-induced migration of LCs *in vitro* and chemotaxis to CCL20/MIP-3$\alpha$ and CCL19/ELC are inhibited by

$DP_1$ activation, without altering expression levels of their receptors CCR6 and CCR7[32]. Similarly, $PGD_2$ and the $DP_1$ agonist BW245C inhibit CCL5/RANTES- and CCL19/ELC-induced chemotaxis of monocyte-derived DCs (MoDCs). Unlike LCs, incubation of MoDCs in the presence of $PGD_2$ alters their expression of cell surface markers and costimulatory molecules, notably decreasing CCR7 and subsequent ability to migrate towards CCL19/ELC[8,34]. $PGD_2$ treatment of MoDCs also impacts their cytokine expression profile, consistently suppressing levels of secreted Th1-related factors IL-12 and CXCL10/IP-10 and enhancing IL-10 production, while more variably affecting Th2-related chemokine expression[8,34]. MoDCs differentiated in the presence of $PGD_2$ have a diminished ability to promote allergen-induced T-cell proliferation, but favor the production of Th2 cells over Th1 cells *in vivo*[8,34]. These functions are replicated in part by selective $DP_1$ agonism with BW245C and not reproduced with $DP_2$ agonist, but some functions such as alteration of cell surface markers appear to be induced by $PGD_2$ alone, implying that $PGD_2$ may also affect DCs in a $DP_1$-independent manner. Collectively, these data indicate a role for $PGD_2/DP_1$ in suppressing the migration of DCs out of tissues to the draining lymph nodes and in favoring Th2 responses.

## THE $LTB_4$ RECEPTORS $BLT_1$ AND $BLT_2$

Leukotriene $B_4$ ($LTB_4$) has been known to induce numerous inflammatory functions in leukocytes, including chemotaxis, degranulation, and adhesion to endothelial surfaces. $BLT_1$, the first described $LTB_4$ receptor, was identified using a cDNA subtraction method in retinoic acid–differentiated HL-60 human leukemia cells[35], with the mouse ortholog being independently identified soon afterwards[36]. $BLT_1$ is a seven-pass transmembrane G-protein coupled receptor with 78% homology at the amino acid level in both the human and mouse forms[37], implying conserved functions across the two species. $BLT_1$ binds $LTB_4$ with high affinity, with $K_d$ ranging from 0.39 to 1.5 n*M* in human neutrophils and transfected CHO and HEK cells[37], and high specificity, as competitive binding studies show displacement only with 20-hydroxy $LTB_4$ and 12-epi $LTB_4$[38]. Initial Northern blotting studies of human tissues displayed highest $BLT_1$ expression in peripheral blood leukocytes, with lower expression in the spleen and thymus[35]. Protein expression of $BLT_1$ on resting human peripheral blood leukocytes has since been demonstrated on granulocytes, monocytes, CD4 and CD8 lymphocytes, $CD19^+$ B cells[39,40,41], as well as on transformed cells, such as certain B-cell leukemia clones[40] and the Jurkat T-cell and HMC-1 mast cell lines[42]. Northern blotting of mouse tissues revealed similar $BLT_1$ expression in IL-5–treated eosinophils, T-cell lymphomas, and activated macrophages and neutrophils, as well as low levels in the lung, lymph nodes, and spleen[43]. While $BLT_1$ expression has been classically restricted to leukocytes, it has recently been described in human endothelial cells[44], and human and mouse vascular smooth muscle cells[45,46] with $BLT_1$ activation resulting in cell migration, proliferation, and chemokine production that contribute to enhanced atherogenic and inflammatory responses (Table 2).

Previous studies had suggested that there existed two separate $LTB_4$ receptors on neutrophils, one binding $LTB_4$ with high affinity and the other binding with lower affinity, with each receptor postulated as mediating separate functions, such as chemotaxis and degranulation[47]. Indeed, a second low-affinity $LTB_4$ receptor, designated $BLT_2$, was identified in humans and mice based on identification of an open reading frame encoding a seven transmembrane-spanning receptor with sequence similarities to $BLT_1$[48], which was independently identified and cloned by other groups[49,50,51]. HEK cells transfected with human $BLT_2$ bind $LTB_4$ with a $K_d$ of 23 n*M*, which is 20 times higher than that of human $BLT_1$[38]. Human $BLT_2$ also binds and is activated by a broader range of eicosanoids, including 20-hydroxy $LTB_4$, 12-epi-$LTB_4$, 12(S)- and 15(S)-hydroxyeicosatetraenoic acid (HETE), and 12(S)- and 15(S)-hydroperxyeicosatetraenoic acid

**TABLE 2**
**$BLT_1$ and $BLT_2$ Receptors**

| | $BLT_1$ | $BLT_2$ |
|---|---|---|
| Receptor expression on leukocytes | *Human*: neutrophils, eosinophils, basophils, mast cells, monocytes, CD4 and CD8 cells<br>*Mouse*: neutrophils, eosinophils, mast cells, monocytes, dendritic cells, activated Th1 and Th2 cells, effector CD8 cells | *Human*: neutrophils, eosinophils, monocytes, HMC-1 cells (mast cell line), dendritic cells<br>*Mouse*: mast cells |
| Agonists | $LTB_4$, 12-epi-$LTB_4$ | $LTB_4$, 12-epi-$LTB_4$, 12(S)-HETE, 12(S)-HPETE, 12(R)-HETE, 20-hydroxy-$LTB_4$ |
| Antagonists | CP105696<br>ZK158252<br>CP195543<br>U75302<br>ONO4057 | LY255283<br>ZK158252<br>CP195543 |

(HPETE)[38]. CHO cells transfected with human $BLT_2$ and stimulated with $LTB_4$ demonstrate calcium flux and chemotaxis inhibitable by pertussis toxin[38,48], indicating function as a classical chemoattractant receptor.

Human $BLT_2$ shares 45.2 and 44.6% homology at the amino acid level with human and mouse $BLT_1$, respectively, while human and mouse $BLT_2$ share an even higher degree of homology at 92.7%[48]. Interestingly, the open reading frame of human $BLT_2$ overlaps with the putative promoter region of the $BLT_1$ gene[52], initially suggesting that transcription of the two receptors may be linked. However, $BLT_1$ expression is largely limited to leukocytes, while human $BLT_2$ is ubiquitously expressed in various tissues, with high levels in the spleen, liver, leukocytes, ovary, testes, pancreas, and heart[48,49,50,51]. At the RNA level, human $BLT_2$ is found in resting CD4, CD8, CD19, and CD14 cells[53]. In spite of significant shared homology, mouse $BLT_2$ differs significantly from human $BLT_2$ in that it is not ubiquitously expressed, but rather has a more restricted distribution in the small intestine, colon, and keratinocytes[54] (Table 2).

## $BLT_1$ and Neutrophils

$LTB_4$ was first recognized as a potent stimulator of neutrophil chemotaxis and aggregation. Since then, multiple roles for $LTB_4$-mediated neutrophil effects have been described, including increased expression of surface neutrophil CD11/CD18 leading to enhanced adhesion to endothelial surfaces[55], induction of granular content release, generation of reactive oxygen species, and retardation of apoptosis[56]. In human cells, $BLT_1$ was first identified on granulocyte-like differentiated HL-60 cells[35], and protein expression was later confirmed using $BLT_1$-specific monoclonal antibodies[57] and presumed to mediate known $LTB_4$ effects on neutrophils. Some uncertainty remains as to the presence of $BLT_2$ in human neutrophils, as some studies demonstrated its presence using quantitative PCR[49] and flow cytometry[43], while a more recent study did not find significant message for $BLT_2$[58].

Mice overexpressing the human $BLT_1$ receptor demonstrate enhanced neutrophil migration in models of $LTB_4$-induced skin inflammation and ischemia perfusion[59]. *In vitro* studies utilizing the $BLT_1$-specific inhibitor CP-105,696 have demonstrated that $LTB_4$ activation of human neutrophils are mediated through $BLT_1$, including calcium flux, chemotaxis, CD11b up-regulation, and retardation of apoptosis[60,61]. However, $BLT_1$ inhibition using U75302 demonstrates residual calcium flux and degranulation when human neutrophils are similarly stimulated with $LTB_4$, suggesting some $BLT_2$

involvement in these processes[43]. Treatment of human peripheral neutrophils with dexamethasone enhances $BLT_1$ expression, as well as calcium flux, chemotaxis, and survival[62]. Treatment with both the $BLT_1$ inhibitor U75302 and $BLT_2$ inhibitor LY255283 blocks these effects[62], implying that endogenous production of $LTB_4$ is critical for these dexamethasone-mediated effects and suggesting an active role for both $BLT_1$ and $BLT_2$ on neutrophil function. Stimulation by other mediators may also lead to activation of neutrophil $LTB_4$ synthesis and autocrine activation, as PAF-induced neutrophil degranulation and chemotaxis are both reduced by inhibition of 5-lipoxygenase activating protein (FLAP) and $BLT_1$[63].

CP-105,696 is also a functional specific inhibitor of murine $BLT_1$[64] and has been used to probe $BLT_1$ function in mouse models of inflammation. Administration of this inhibitor in a mouse cremasteric venule model of inflammation decreases neutrophil adhesion to and transmigration through IL-1β–stimulated endothelium[65], suggesting a role for $BLT_1$ in neutrophil extravasation. CP-105,696 also reduces neutrophil migration in an $LTB_4$-mediated model of skin inflammation[66] and synovial neutrophil infiltration in autoantibody-induced inflammatory arthritis[67]. In a model of sublethal cecal puncture, treatment of mice with CP-105,696 reduces neutrophil migration into the peritoneal cavity with subsequent increased bacterial loads and increased mortality[68]. In a rat model of renal ischemia-reperfusion injury, ONO-4057, a $BLT_1$/$BLT_2$ inhibitor, preserves renal function and reduced kidney neutrophil infiltration and structural damage[69].

The generation of $BLT_1^{-/-}$ mice has also highlighted the necessity for $BLT_1$ in directing neutrophil migration in numerous inflammatory responses. Initial characterization of neutrophils isolated from $BLT_1^{-/-}$ mice displayed eradication of calcium flux, chemotaxis[70,71], and degranulation[72] in response to $LTB_4$, as well as decreased early neutrophil recruitment in zymosan-induced peritonitis[71]. In an OVA-induced model of asthma, there are reduced numbers of neutrophils in the BAL after early antigen challenge in $BLT_1^{-/-}$ mice compared with wild-type, and $BLT_1^{-/-}$ mice also display diminished neutrophil recruitment in the airways after administration of OVA-IgG1 and OVA-IgE complexes[73]. In an autoantibody-induced model of inflammatory arthritis, $BLT_1^{-/-}$ mice are resistant to the development of arthritis, but adoptive transfer of wild-type neutrophils into these mice restores the arthritic response, a finding also replicated by transferring wild-type neutrophils into 5-LO–deficient mice that cannot generate $LTB_4$[67,74]. These studies show that autocrine activation of $BLT_1$ on neutrophils is required for $BLT_1$-deficient neutrophils to be recruited into the joint subsequently, indicating a novel mechanism of autocrine $BLT_1$-mediated neutrophil activation in the amplification of innate immune responses.

## $BLT_1$ and Eosinophils

$LTB_4$ has numerous stimulatory effects on eosinophils, including the induction of chemotaxis, aggregation[75], degranulation[76], and respiratory burst[77], although it does not have any effect on eosinophil apoptosis *in vitro*[61]. Since then, both $BLT_1$ and $BLT_2$ mRNA have been detected in human eosinophils[49], indicating that either of these receptors may mediate these actions. CP-105,696–mediated $BLT_1$ inhibition diminishes $LTB_4$-induced calcium flux in human eosinophils[61]; however, further detailed studies of the differential roles of $BLT_1$ and $BLT_2$ activation on human eosinophil chemotaxis, aggregation, and degranulation have not yet been described in the literature. Of note, basophils also express $BLT_1$, but not $BLT_2$, and IL-3-primed basophils degranulate upon exposure to $LTB_4$[58].

Murine $BLT_1$ was first identified using degenerate PCR amplification of eosinophil mRNA with human $BLT_1$ expression being confirmed on human eosinophils[36], and accordingly, abrogation of $BLT_1$ function has been shown to lead to attenuated eosinophil migration in various mouse models of inflammation. $BLT_1^{-/-}$ mice have preferentially decreased eosinophil recruitment in thioglycollate-induced peritonitis[70] and in early antigen challenge in OVA-induced asthma[73]. In addition, administration of CP-105,696 reduces eosinophil infiltration of the spinal cord after induction of experimental allergic encephalomyelitis (EAE)[78]. CP-105,696 diminishes eosinophil recruitment in models of stem cell factor (SCF)- and OVA-induced pleurisy in mice[79]. It was thought that SCF

stimulates mast cells to produce $LTB_4$, leading to subsequent eosinophil $BLT_1$-mediated migration. However, eosinophil recruitment after direct injection of CCL11/eotaxin-1 into the pleural space is also diminished by $BLT_1$ inhibition, although the CCL11/eotaxin-1 injection itself does not increase pleural $LTB_4$ levels[80], leading the authors to postulate that CCL11/eotaxin-1 induces $BLT_1$-$LTB_4$ autocrine activation of eosinophils at a microscopic level necessary for their migration towards CCL11/eotaxin-1 and possibly other chemoattractants as well.

## $BLT_1$ and $BLT_2$ and Mast Cells

An analysis of mast cell–derived factors that induce chemotaxis of immature murine bone marrow–derived mast cells (BMMCs) identified $LTB_4$ as the active chemotactic agent[81]. However, as they mature in SCF, both murine BMMCs and human cord blood–derived mast cells lose their ability to migrate towards $LTB_4$[81], suggesting a mechanism by which mature mast cells recruit their progenitors from the bone marrow to tissue sites to maintain their presence in the periphery. In this study, levels of $BLT_1$ mRNA decreased with murine mast cell maturation and were presumed to be the cause for decreased ability to migrate towards $LTB_4$, but the specific $LTB_4$ receptor responsible for mast cell chemotaxis was not confirmed through the use of pharmacologic inhibitors. Another study has subsequently demonstrated message for both $BLT_1$ and $BLT_2$ in murine BMMCs and the human mast cell line HMC-1[42]. Increasing the time of incubation in SCF diminishes human mast cell $BLT_1$ surface expression, suggesting an explanation for the loss of migration to $LTB_4$ during the maturation of murine BMMCs. However, chemotaxis of both murine BMMCs and HMC-1 cells towards $LTB_4$ is reduced with either $BLT_1$ or $BLT_2$ inhibition[42], indicating that both these receptors are capable of inducing chemotaxis in mast cells. Although the physiologic significance of this dual receptor usage on mast cells is not yet known, $LTB_4$ clearly has the potential of contributing to allergic and inflammatory responses by recruiting mast cells to tissue sites during inflammation and maintaining tissue mast cell homeostasis after preexisting mast cells have released their granular contents.

## $BLT_1$ and Monocytes

$LTB_4$ has been shown to activate monocytes and macrophages by inducing chemotaxis, surface CD11b expression, and phagocytosis. Message for both $BLT_1$ and $BLT_2$ receptors has been detected in $CD14^+$ monocytes[53], and flow cytometric studies of human peripheral blood leukocytes have confirmed $BLT_1$ expression on $CD14^{++}CD16^-$ monocytes, which represent 85–90% of the peripheral monocyte population[82]. Both $BLT_1$ expression and activity are decreased by the addition of inflammatory cytokines such as IFN-γ and TNF, while $BLT_1$ transcription is elevated by IL-10 and dexamethasone[82]. $BLT_1^+$ monocytes also express increased levels of CCR2, a chemokine receptor whose expression is also regulated similarly to $BLT_1$, implying a cooperative role for $BLT_1$- and CCR2-mediated signaling and chemotaxis in this inflammatory monocyte subset. Additionally, $BLT_1$-activated monocytes produce CCL2/MCP-1[83]. These data suggest that $BLT_1$-stimulated monocytes may amplify further monocyte recruitment by generating CCL2/MCP-1 and promoting subsequent CCR2-mediated chemotaxis.

In mice, $BLT_1$ and CCR2 signaling on monocytes also appear to be linked. In a cecal ligation and puncture (CLP) model of septic peritonitis, CCL2/MCP-1 blockade reduces neutrophil and macrophage infiltration and $LTB_4$ production; however, $BLT_1$ inhibition with CP-105,696 also reduces neutrophil and macrophage infiltration and CCL2/MCP-1 production, demonstrating cross-talk between the two chemoattractants in inducing inflammatory responses to infection, as inhibition of either CCL2/MCP-1 or $BLT_1$ significantly decreased mouse survival[84]. CP-105,696 also inhibits monocytic foam cell migration and CD11b expression in a atherosclerosis model[85], and nitric oxide production in *Trypanosoma cruzi*-infected macrophages[86].

## $BLT_1$ and T Lymphocytes

Although $LTB_4$ is characteristically identified as a potent activator of leukocytes of the innate immune system, such as granulocytes and monocytes, $LTB_4$ has been previously shown to bind to T cells[87] and induce T-cell chemotaxis *in vitro*[88], as well as T-cell cytokine production[37]. Characterization of the $BLT_1$ receptor led to an exploration of its expression on human peripheral blood leukocytes and its identification on T cells[39,53]. $BLT_1$ is also expressed in murine T-cell lymphomas[36], suggesting that it may be expressed on peripheral blood T cells in mice as well.

Exploration of the functional roles of $BLT_1$ on T cells using mouse models of inflammation has revealed that $BLT_1$ activation plays a critical role in the recruitment of effector CD4 and CD8 cells early in inflammation. Although $BLT_1$ is not found in naïve T cells, $BLT_1$ expression is significantly up-regulated in activated CD4 Th1 and Th2 cells[89], and effector CD8+ cells[90,91]. In the early phases of an active immunization asthma model, $BLT_1^{-/-}$ mice exhibit defective recruitment of CD4 and CD8 cells in the airways, but have normal levels of airway T cells in an adoptive transfer model, indicating an important role for $BLT_1$-mediated T-cell trafficking in both humoral and adaptive immune responses[89]. Peribronchial lymph node cells from OVA-sensitized $BLT_1^{-/-}$ mice have impaired Th2 cytokine (IL-5 and IL-13) production, ultimately leading to diminished airway hypersensitivity, airway and lung eosinophilia, goblet cell hyperplasia, and IgE production after OVA inhalation[72]. These studies demonstrate that $BLT_1$ activation of helper T cells is a critical component of the early allergic response.

Effector CD8 cells also display significantly higher levels of $BLT_1$ expression compared with central memory CD8 cells and migrate in response to $LTB_4$ *in vitro* and *in vivo* settings[90,91]. Analysis of mast cell supernatants identify $LTB_4$ to be the major mast cell–produced chemotactic factor inducing effector CD8 T cell migration[91], and that mast cell- and $Fc\varepsilon RI^{-/-}$ mice produce lower levels of $LTB_4$ in BAL fluid after OVA sensitization and challenge[92]. Transfer of OVA-primed $BLT_1^{-/-}$ effector CD8 T cells into sensitized $CD8^{-/-}$ mice fails to restore airway hyper-responsiveness (AHR), eosinophilic inflammation, and IL-13 production both in an adoptive transfer and a mast-cell dependent model of allergic airway disease[92,93]. Collectively, these studies place an important role for $BLT_1$ activation of T cells on the generation of allergic airway disease, both in the induction of Th2 cell responses with IgE and cytokine production, and in $T_{EFF}$ recruitment and activation, providing a critical transition between the initial rapid innate immune responses towards the development and propagation of adaptive immunity.

Other clinically relevant, T-cell–mediated inflammatory processes are dependent on $BLT_1$. $BLT_1$ blockade with CP-105,696 in a murine heterotopic cardiac transplantation model increases allograft survival with diminished T lymphocyte, granulocyte, and macrophage tissue infiltration into affected organs[94]. In a murine acute lung rejection model, adoptive transfer of lung-specific $BLT_1^{-/-}$ CD8 effector cells results in diminished lung inflammation and increased mouse survival, and $BLT_1^{-/-}$ mice that undergo tracheal transplants display significantly less fibroproliferation and airways hyper-responsiveness, along with defective T-cell recruitment[95]. In a mouse model of autoimmune uveitis, adoptive T-cell transfer of antigen-specific $BLT_1^{-/-}$ T cells results in less severe disease than transfer of T cells from wild-type mice, although there appears to be a $BLT_1$-dependent contribution to the recruitment of other immune cells such as neutrophils[96].

A detailed characterization of $BLT_1$ in human resting peripheral blood cells has identified $BLT_1$ expression on a rare subset of $CD3^+CD4^+$ and $CD3^+CD8^+$ cells (0–1%)[41]. This $BLT_1^+$ population is enriched for late activation markers CD38 and HLA-DR and inflammatory chemokine receptors, and they tend to express more polarizing cytokines. However, dendritic cell stimulation of CD4 and CD8 cells may lead to a dramatic increase in surface $BLT_1$ expression. Asymptomatic atopic asthmatics demonstrate increased numbers of $BLT_1^+$ CD4 and CD8 memory T cells in the airways, and EBV-specific CD8 cells from acutely infected individuals also have increased levels of $BLT_1$ expression compared with those with asymptomatic chronic infection[41]. In addition, patients with bronchiolitis obliterans post lung transplantation express greater numbers of $BLT_1^+$ CD4 and CD8 cells in the airways compared with normal transplanted lungs[95]. These studies demonstrate that $BLT_1$ is constitutively expressed on antigen-primed memory/effector T cells whose phenotype suggests a primary function of responding rapidly to inflammatory stimuli. In the setting of infection or T-cell activation, however, $BLT_1$ expression is transiently increased on this T-cell subpopulation, enhancing their ability to quickly respond to the early phases of infectious and inflammatory processes.

## $BLT_1$ and $BLT_2$ and Dendritic Cells

Murine bone marrow–derived dendritic cells (mBMDCs) migrate in response to $LTB_4$, an effect abrogated in cells lacking $BLT_1$[97]. In addition, $LTB_4$ up-regulates expression of CCR7 and its ligand CCL19/ELC, both of which mediate mBMDC migration to the draining lymph nodes. Accordingly, $BLT_1^{-/-}$/$BLT_2^{-/-}$ double deficient mice develop diminished dendritic cell migration to the lymph node, and ear inflammation and swelling in a model of contact hypersensitivity[97]. These data indicate that $BLT_1$ activation of murine dendritic cells contributes to the inflammatory response by enhancing their migration via CCR7 and its ligands to the draining lymph nodes, where they encounter and activate antigen-specific cells. Human monocyte-derived dendritic cells also migrate towards $LTB_4$ in a PTX-sensitive fashion. However, these cells have been shown to express $BLT_2$ message and chemotaxis is eliminated by the $BLT_2$-specific inhibitor LY255283[98]. These studies confirm an active biological role for $LTB_4$ activation in dendritic cell function, although it appears in these initial studies that there may be differences in receptor usage between species, which need to be further defined.

## THE CYSTEINYL LEUKOTRIENE RECEPTORS $CYSLT_1$ AND $CYSLT_2$

The cysteinyl leukotrienes (cysLTs), $LTC_4$, $LTD_4$, and $LTE_4$, are rapidly synthesized by macrophages, mast cells, eosinophils, and basophils, and induce critical components of the allergic response, including bronchial smooth muscle constriction, vascular permeability, and leukocyte activation[99]. The cysLTs mediate their actions through two receptors, $CysLT_1$ and $CysLT_2$, both seven transmembrane-spanning G-protein receptors. Human $CysLT_1$ was independently cloned and characterized by two groups[100,101]. $CysLT_1$ binds preferentially to $LTD_4$, with 200-fold less affinity for $LTE_4$ and $LTC_4$, and cellular calcium mobilization by $CysLT_1$ is not affected by PTX treatment. However, previous studies with THP-1 cells have suggested that while calcium flux is mediated by a PTX-insensitive signaling pathway, chemotaxis is mediated through a separate, PTX-sensitive mechanism[102]. Northern blot analysis detects $CysLT_1$ message rather ubiquitously in human organ tissues, with highest expression in the spleen and peripheral blood leukocytes, followed by the lung, placenta, and intestine, with detectable transcript in the promyelocytic cell line HL-60 and the lymphocytic leukemia cell line U937[100,101]. *In situ* hybridization studies have demonstrated human $CysLT_1$ in lung smooth muscle and macrophages, eosinophils, monocytes, and B cells[103], and $CysLT_1$ message and signaling are present in human mast cells derived from cord blood as well[104]. $CD34^+$ hematopoietic progenitor cells also express $CysLT_1$ and migrate in response to $LTD_4$[105]. Transgenic mice overexpressing human $CysLT_1$ develop enhanced airway eosinophilia, Th2 cytokine production, and airways hyper-responsiveness in a model of *Aspergillus fumigatus* sensitization and challenge[106]. Mouse $CysLT_1$ bears 87% homology with the human ortholog at the amino acid level and shares similar binding characteristics[107,108,109]. Mice express $CysLT_1$ at high levels in the skin, lung, macrophage, and small intestine, although some strain difference exists with more intense skin expression in C57Bl/6 mice compared with the 129+Ter/Sv strain[109]. Detailed *in situ* hybridization of 129+Ter/Sv mouse skin reveals $CysLT_1$ in subcutaneous connective tissue fibroblasts[109] (Table 3). $CysLT_1$ blockade with the agents montelukast, zafirlukast, and pranlukast has significant relevance in the management of allergic respiratory diseases, as they have consistently been shown to be effective in reducing airways hyper-responsiveness and eosinophilia, and improving symptoms and quality of life in patients with asthma and allergic rhinitis[99,110].

**TABLE 3**
**$CysLT_1$ and $CysLT_2$ Receptors**

| | $CysLT_1$ | $CysLT_2$ |
|---|---|---|
| Receptor expression on leukocytes | *Human*: eosinophils, basophils, mast cells, monocytes and macrophages, dendritic cells, B cells, T cells<br>*Mouse*: macrophages | *Human*: eosinophils, mast cells, macrophages, dendritic cells, T cells<br>*Mouse*: macrophages |
| Agonists | $LTD_4$, $LTC_4$, $LTE_4$ | $LTC_4$, $LTD_4$, $LTE_4$ |
| Antagonists | Montelukast (MK476)<br>Zafirlukast (ICI204219)<br>Pranlukast (ONO1078)<br>MK571<br>BAYu9773<br>LY171833 | BAYu9773 (partial agonist) |

Human $CysLT_2$ shares 38% amino acid homology with human $CysLT_1$, and binds $LTC_4$ and $LTD_4$ with equal affinity, followed by $LTE_4$[111,112,113]. Northern Blot and dot blot analysis has detected high levels of human $CysLT_2$ in the heart, spleen, peripheral blood leukocytes, placenta, and adrenal glands, while *in situ* hybridization demonstrates expression in lung smooth muscle cells, adrenal medulla, and cardiac Purkinje fibers[111,112,113]. In addition, $CysLT_2$ message has been identified in HUVECs, levels of which are elevated on IL-4 stimulation or upon reaching confluence[114]. Among leukocytes, human $CysLT_2$ is expressed on macrophages, eosinophils[111], and mast cells[115]. Mouse $CysLT_2$ shares 73% sequence homology with human $CysLT_2$ and 39% homology with mouse $CysLT_1$, and binds to the same natural ligands as human $CysLT_2$ with similar affinities[109,116]. Mouse $CysLT_2$ transcript is more ubiquitously distributed, including brain, lung, spleen, small intestine, kidney, skin, adrenals, thymus, and spinal cord, while cellular expression has been described in macrophages and subcutaneous fibroblasts[109,116] (Table 3).

## $CysLT_1$ and Eosinophils

The presence of CysLTs in allergic and atopic conditions characterized by eosinophilic inflammation suggests that they may play a direct role in inducing eosinophil activation and migration. Mice deficient in $LTC_4$ synthase, the enzyme required for CysLT synthesis, have markedly decreased eosinophilic inflammation in an asthma model[117], and selective $CysLT_1$ inhibition reduces tissue eosinophilia in both murine[118] and human allergic conditions. *In vitro* studies have shown that $LTC_4$, $LTD_4$, and $LTE_4$ induce surface expression of the adhesion molecule Mac-1 (but not LFA-1) and chemotaxis of human peripheral blood eosinophils in a $CysLT_1$-dependent manner[119,120]. $CysLT_1$ inhibition also diminishes eosinophil migration across endothelial cell layers in a static *in vitro* model[121]. These studies suggest that one mechanism by which CysLTs contribute to eosinophilic inflammation is by directly recruiting eosinophils from the circulation into target organs.

However, other proinflammatory effects of $CysLT_1$ activation on eosinophils include promotion of their survival and effector functions. $LTD_4$ enhances eosinophil differentiation from progenitor cells[122] and promotes mature eosinophil survival[123], of which both processes are $CysLT_1$ dependent. CysLTs also induce eosinophil granule release. In a murine asthma model, montelukast inhibits eosinophil degranulation in lung tissues[118]. Eosinophils differentiated *in vitro* from human cord blood release preformed IL-4 after CysLT stimulation in a dose- and PTX-dependent manner, a process blocked by $CysLT_1$ inhibition[124]. When peripheral blood eosinophils are used, however, $LTC_4$ and $LTD_4$ elicit IL-4 release when added to permeabilized eosinophils only, while addition of $CysLT_1$ and $CysLT_2$ inhibitors does not block this release. These findings suggest that in addition to binding $CysLT_1$, CysLTs may bind novel CysLT intracellular receptors necessary for the transcription and release of inflammatory cytokines[125].

## $CysLT_1$ and Monocytes/Macrophages

Initial characterization of the human $CysLT_1$ receptor revealed prominent staining in pulmonary macrophages[103], suggesting a potential important role for CysLT activation of the monocyte/macrophage lineage in allergic airway disease. In a rat alveolar macrophage cell line NR8383, combined treatment with $LTD_4$ and lipopolysaccharide (LPS) significantly increases macrophage production of CCL3/MIP-1$\alpha$, a chemoattractant for eosinophils and monocytes, a response reversible by $CysLT_1$ inhibition[126]. In this fashion, CysLT activation of macrophages serves as an additional means of inducing eosinophilic inflammation. Of interest is that both $LTD_4$ and LPS were required for this response, implicating a role for Toll-like receptors in the initiation of allergic responses predominated by CysLTs.

Message for $CysLT_1$ is found in resting human monocytes and macrophages, as well as the monocytic cell line THP-1. Incubation with the Th2-like cytokines IL-4 and IL-13 further increases $CysLT_1$ transcription, surface expression, and chemotaxis towards $LTD_4$, overall enhancing monocyte participation in the allergic response[127]. IL-4 stimulation of THP-1 cells induces $CysLT_1$ expression mediated by STAT6 activation, leading to the enhanced production of CCL2/MCP-1[128]. In addition to mediating monocyte chemotaxis towards CysLTs directly, $CysLT_1$ may enhance their chemotaxis towards other chemoattractants. Addition of zafirlukast or montelukast to THP-1 cells or human monocytes reduces chemotaxis and calcium flux in response towards CCL2/MCP-1 through a p38-MAPK pathway, without altering their production of $LTB_4$ or $LTD_4$[129]. These data suggest that $CysLT_1$ activation of monocytes amplifies Th2-mediated responses, in which Th2 cytokines induce enhanced monocyte responsiveness to $LTD_4$ and promote the recruitment of monocytes and eosinophils towards sites of inflammation.

## $CysLT_1$ and $CysLT_2$ and Mast Cells

Mast cells are an integral component of allergic and inflammatory responses, and produce CysLTs on stimulation with IL-4 or Fc$\varepsilon$RI cross-linking. Both $CysLT_1$ and $CysLT_2$ have been identified in cord blood–derived human mast cells and the human mast cell line HMC-1[115,130,131]. $CysLT_1$ activation increases human and mouse mast cell proliferation in the presence of IL-4[132]. Unprimed human mast cells respond to $LTC_4$ and $LTD_4$ as demonstrated by calcium mobilization, a process primarily mediated through $CysLT_1$. $LTC_4$ and $LTD_4$ also amplify the transcription and release of IL-5, TNF-$\alpha$, and CCL4/MIP-1$\beta$ through ERK- and NFAT/calcineurin-mediated $CysLT_1$ activation of IL-4-primed mast cells. Mast cell cytokine production resulting from IgE cross-linking of Fc$\varepsilon$RI is also partially dependent on autocrine production of CysLTs and subsequent activation of $CysLT_1$. However, mast cell degranulation and production of histamine and $PGD_2$ is not affected by $CysLT_1$ activation[104]. These studies show a role for $CysLT_1$ activation of mast cells in amplifying Th2 inflammation primarily through activating the transcription and release of cytokines and chemokines, rather than inducing immediate degranulation.

A GenBank search for sequences homologous with $CysLT_1$ and $CysLT_2$ revealed that these receptors share 24–32% homology with the purinergic (P2Y) receptor family, and in particular, with the P2Y6 receptor, which preferentially binds the pyrimidinergic ligand UDP. Indeed, mast cell $CysLT_1$ also acts as an active receptor for UDP, as shown by initiation of calcium flux that is blocked by the $CysLT_1$ inhibitor MK571[130]. UDP activation of $CysLT_1$ strongly induces the production of TNF-$\alpha$ and CCL4/MIP-1$\beta$ in

IL-4–primed mast cells in a manner similar to $LTD_4$ and $LTE_4$, but it induces IL-5 less strongly than the CysLTs[104].

Priming of mast cells with IL-4 prior to $CysLT_1$ stimulation increases calcium mobilization, but it does not affect $CysLT_1$ receptor cell surface expression[130]. $CysLT_2$ receptor expression is increased after IL-4 priming, however, accounting for this difference and indicating that this is a functional receptor on mast cells. $CysLT_2$ regulation and signaling on mast cells also differs from $CysLT_1$ in other ways. Stimulation with $LTC_4$ and $LTD_4$ in the presence of $CysLT_1$ inhibitor suppresses the production of most cytokines, with the exception of CCL2/MCP-1 and CXCL8/IL-8, indicating that these are induced by $CysLT_2$ activation alone. Unlike $CysLT_1$, which has been shown to signal through PTX-insensitive pathways, PTX completely blocks $CysLT_2$-mediated CXCL8/IL-8 production, a neutrophil-active chemokine[115]. Therefore, $CysLT_2$ may play a role in innate immune responses by activating mast cell production of a distinct set of chemokines.

## $CysLT_1$ and Dendritic Cells

An early study demonstrated a direct role for CysLTs in controlling dendritic cell migration through studies of multidrug resistance associated protein 1 (MRP1, also known as Abcc1), which is required for CysLT transport outside of the cell[133]. In a skin inflammation model, dendritic cells of MRP1/Abcc1$^{-/-}$ mice are retained in the epidermis and dermis with diminished migration to the draining lymph nodes. In addition, chemotaxis towards CCL19/ELC is dramatically decreased by 90% in MRP1/Abcc1$^{-/-}$ bone marrow–derived dendritic cells, with 35% reduction in chemotaxis to CCL21/SLC. Administration of exogenous $LTD_4$ either *in vivo* or *in vitro* reinstates the dendritic cell chemotactic response, indicating that dendritic cells must synthesize $LTD_4$ and export it out of the cell, where it binds to $CysLT_1$ receptor in an autocrine manner and induces migration towards CCL19/ELC.

$CysLT_1$ and $CysLT_2$ receptors have subsequently been identified on human MoDCs, although *in vitro* differentiation and maturation results in a relative down-regulation of CysLT receptor expression with resultant reduced response to $LTD_4$ as manifested by decreased calcium flux and chemotaxis[134]. $CysLT_1$ antagonism has been shown to have significant effects on dendritic cell localization in the early asthma response, as pranlukast-treated patients have fewer circulating myeloid CD33$^+$ DCs compared with placebo-treated controls, although there are no differences in the number of circulating plasmacytoid CD123$^+$ DCs[135]. This difference may be accounted for by the fact that myeloid DCs express higher levels of $CysLT_1$ than plasmacytoid dendritic cells and would be affected more by $CysLT_1$ inhibition[135], but this decrease in circulating myeloid DCs after pranlukast treatment does not definitively indicate between which compartments dendritic cell migration route is interrupted. $CysLT_1$ activation of DCs also induces cytokine production, particularly of the Th2 type. Upon allergen stimulation, MoDCs from atopic patients produce more IL-10 and TNF-$\alpha$, and induce CD4 cells to produce more IL-5 and IFN-$\gamma$ when compared with MoDCs from nonatopic controls, processes which are inhibited by pranlukast and montelukast[136]. $CysLT_1$ stimulation of MoDCs also induces CXCL8/IL-8 production, pointing to a role for DCs in stimulating innate immune responses as well[137].

$CysLT_1$ activation of murine dendritic cells also results in a number of proinflammatory effects. In contrast with human DCs, murine bone marrow–derived DCs express more $CysLT_1$ on stimulation with antigen, although this has been identified only at the mRNA level[138]. $CysLT_2$ expression on murine dendritic cells has not yet been described in the literature. In one study, *in vitro* stimulation of murine DCs with both CysLTs and antigen simultaneously results in both IL-10 and IL-12 production; however, while IL-10 production is suppressed by $CysLT_1$ blockade, IL-12 production is increased[138]. These findings suggest that $CysLT_1$ activation of murine dendritic cells primes the Th2 response by promoting the production of Th2-type cytokines. It is interesting to postulate that this observed increase in IL-12, a Th1 cytokine, may be due to unopposed $CysLT_2$ activation of dendritic cell and skewing towards a Th1 response, if $CysLT_2$ is eventually found to exist on these cells. Dendritic cells isolated from the spleens of OVA-sensitized mice generate both Th1 and Th2 cytokines on antigen stimulation *in vitro*, and are able to enhance proliferation of coincubated CD4 cells, processes that are inhibited by treating the mice with pranlukast prior to isolation of dendritic cells[139].

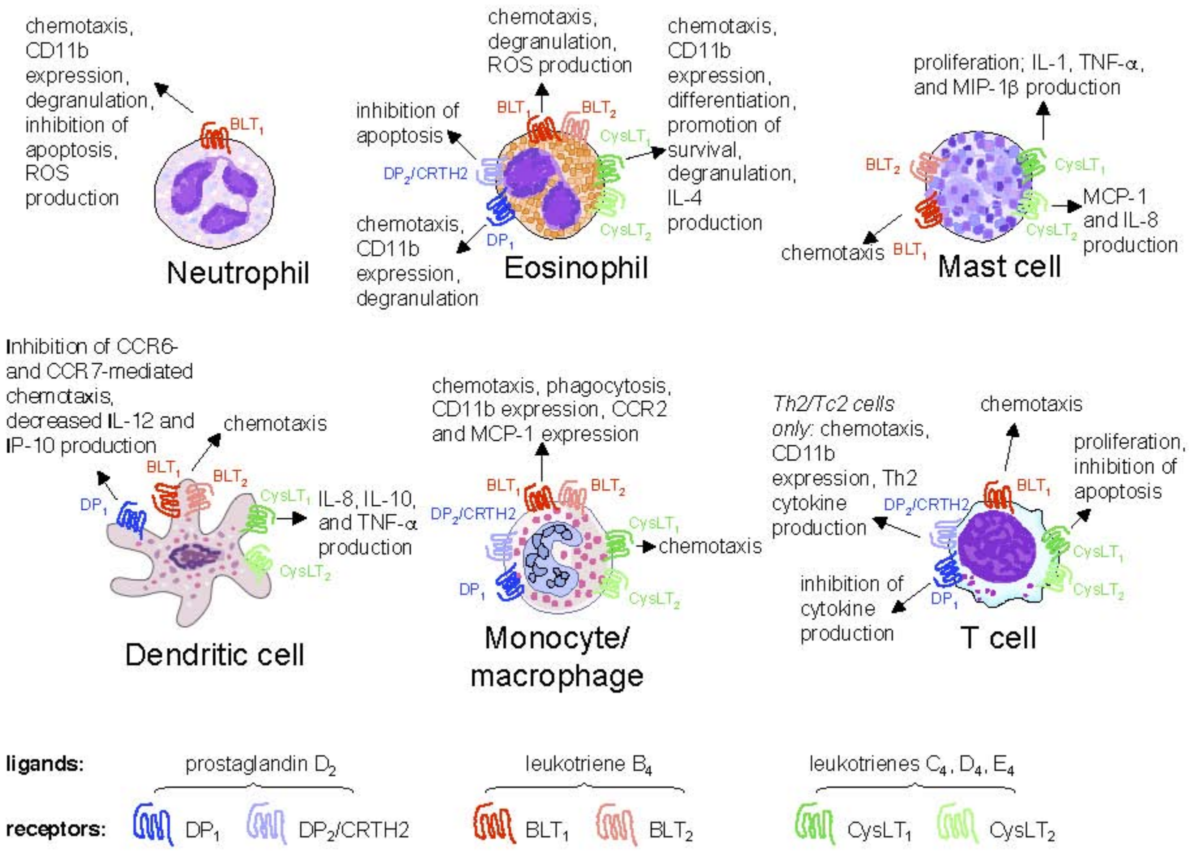

**FIGURE 1.** Distribution and functions of $PGD_2$, $LTB_4$, and CysLT receptors on human leukocyte populations. Leukocyte expression and known functions of $DP_1$, $DP_2$/CRTH2, $BLT_1$, $BLT_2$, $CysLT_1$, and $CysLT_2$ receptors after activation with their respective ligands are depicted. This diagram represents human leukocytes; while mouse and other species have similar distribution and functions, significant differences exist, as described in this review. ROS, reactive oxygen species.

## $CysLT_1$ and Lymphocytes

Resting T cells isolated from the peripheral blood of humans[103] and mice[140] express very low levels of $CysLT_1$ and $CysLT_2$. However, murine $CysLT_1$ expression is induced in *in vitro* polarized Th1 and Th2 cells, and TCR- and CD28-activated bulk CD4 and CD8 cells, along with the ability to migrate to $LTD_4$[140]. One study demonstrated that only 0.7 and 2.5% of resting human T cells express $CysLT_1$ and $CysLT_2$, respectively, but incubation with IL-4 and IL-13 increases $CysLT_1$ and $CysLT_2$ expression to 9.8 and 6.9%, and anti-CD3 antibody increases expression to 78 and 65% of T cells, respectively[141]. Interestingly, incubation of CD3-activated T cells with montelukast decreases proliferation and increases the number of cells undergoing apoptosis[141], implying an important role for autocrine/paracrine activation of the $CysLT_1$ receptor in maintaining the viability of activated T cells. Studies of the CysLT

transporter MRP1/Abcc1 have provided insight into the role of CysLT signaling in murine T-cell movement[142], suggesting that autocrine/paracrine activation by T cells by CysLTs may play a role in CCR7-directed movement into lymph nodes.

Of note, one study has demonstrated that resting B lymphocytes express low levels of $CysLT_1$ and $CysLT_2$, but only $CysLT_1$ expression is increased after stimulation with IL-4 and either anti-CD40 or CD154-transfected fibroblasts. $CysLT_1$ activation by $LTD_4$ also results in increased IgG and IgE production and secretion, thereby providing another means of enhancing Th2-mediated stimulation[143].

## SUMMARY

It has long been recognized that eicosanoids participate in critical physiological functions, such as the regulation of smooth muscle tone, vascular permeability, neuronal function, and platelet aggregation. However, many recent studies have uncovered that they have additional important roles in regulating both innate and adaptive immune responses, and the success of cysteinyl receptor blockade for the treatment of allergic and atopic conditions has demonstrated that inhibiting these pathways are viable therapeutic options for inflammatory diseases. While the numbers of eicosanoid receptors being identified on leukocyte subpopulations continues to grow, their roles in inducing specific immune responses still remain to be fully explicated (Fig. 1). The availability and development of specific eicosanoid receptor inhibitors and future studies in mice genetically deficient in these receptors will help to further characterize their diverse functions in both driving and suppressing these important immune responses.
